# Association of Limited In-Person Attendance in US National Football League and National Collegiate Athletic Association Games With County-Level COVID-19 Cases

**DOI:** 10.1001/jamanetworkopen.2021.19621

**Published:** 2021-08-17

**Authors:** Asmae Toumi, Haoruo Zhao, Jagpreet Chhatwal, Benjamin P. Linas, Turgay Ayer

**Affiliations:** 1 Institute for Technology Assessment, Massachusetts General Hospital, Boston; 2 Georgia Institute of Technology, Atlanta; 3 Harvard Medical School, Boston, Massachusetts; 4 Section of Infectious Diseases, Department of Medicine, Boston Medical Center, Boston, Massachusetts; 5 Department of Epidemiology, Boston University School of Public Health, Boston, Massachusetts

## Abstract

**Importance:**

In 2020 and early 2021, the National Football League (NFL) and National Collegiate Athletic Association (NCAA) opted to host football games in stadiums across the country. The in-person attendance of games varied with time and from county to county. There is currently no evidence on whether limited in-person attendance of games is associated with COVID-19 case numbers on a county-level.

**Objective:**

To assess whether NFL and NCAA football games with limited in-person attendance were associated with increased COVID-19 cases in the counties they were held compared with a matched set of counties.

**Design, Setting, and Participants:**

In this time-series cross-sectional study, every county hosting NFL or NCAA games with in-person attendance (treated group) in 2020 and 2021 was matched with a county that that did not host a game on the corresponding day but had an identical game history for up to 14 days prior (control group). A standard matching method was used to further refine this matched set so that the treated and matched control counties had similar population size, nonpharmaceutical interventions in place, and COVID-19 trends. The association of hosting games with in-person attendance with COVID-19 cases was assessed using a difference-in-difference estimator. Data were analyzed from August 29 to December 28, 2020.

**Exposures:**

Hosting NFL or NCAA games.

**Main Outcomes and Measures:**

The main outcome was estimation of new COVID-19 cases per 100 000 residents at the county level reported up to 14 days after a game among counties with NFL and NCAA games with in-person attendance.

**Results:**

A total of 528 games with in-person attendance (101 NFL games [19.1%]; 427 NCAA games [80.9%]) were included. The matching algorithm returned 361 matching sets of counties. The median (interquartile range [IQR]) number of attendance for NFL games was 9949 (6000 to 13 797) people. The median number of attendance for NCAA games was not available, and attendance was recorded as a binary variable. The median (IQR) daily new COVID-19 cases in treatment group counties hosting games was 26.14 (10.77-50.25) cases per 100 000 residents on game day. The median (IQR) daily new COVID-19 cases in control group counties where no games were played was 24.11 (9.64-48.55) cases per 100 000 residents on game day. The treatment effect size ranged from −5.17 to 4.72, with a mean (SD) of 1.21 (2.67) cases per 100 000 residents, within the 14-day period in all counties hosting the games, and the daily treatment effect trend remained relatively steady during this period.

**Conclusions and Relevance:**

This cross-sectional study did not find a consistent increase in the daily COVID-19 cases per 100 000 residents in counties where NFL and NCAA games were held with limited in-person attendance. These findings suggest that NFL and NCAA football games hosted with limited in-person attendance were not associated with substantial risk for increased local COVID-19 cases.

## Key Points

**Question:**

Are football games with limited in-person attendance associated with increased county-level COVID-19 cases?

**Findings:**

This cross-sectional study of US counties that hosted National Football League and National Collegiate Athletic Association football games used a matching and difference-in-differences design to estimate the association of games with limited in-person attendance with county-level COVID-19 spread. There was no association between higher county-level COVID-19 cases and hosting football games with limited in-person attendance.

**Meaning:**

This study suggests that football games held with limited in-person attendance were not associated with increased COVID-19 cases in the counties they were held.

## Teaser

This cross-sectional study examines the association of limited in-person attendance at US National Football League (NFL) and National Collegiate Athletic Association (NCAA) football games with changes in county-level COVID-19 case numbers.

## Introduction

The Centers for Disease Control and Prevention has advised that mass in-person events have the potential for substantial spread of COVID-19.^1^ To curb the spread of COVID-19, states have implemented nonpharmaceutical interventions with varying intensity, including closure of work places, limit on indoor and outdoor gatherings, and travel restrictions.^2^ Of note, sporting events have been banned or cancelled owing to the potential of mass gatherings to become super-spreader events.^3^

In early 2020, the National Basketball Association and National Hockey League temporarily suspended their 2019 to 2020 seasons in an effort to limit the spread of COVID-19. A few months later, both leagues committed to resuming games in a “bubble” format, in which games were held in select sites with no fan attendance. The National Basketball Association suspended their season a second time owing to a strike, while the National Hockey League resumed their season with no interruption. In the late summer of 2020, the National Football League (NFL) and National Collegiate Athletic Association (NCAA) made the decision to play games in their respective stadiums, with many hosting in-person attendance in a limited capacity. Despite various restrictions in place on sporting and nonsporting events, COVID-19 cases continued to increase nationally from 4.62 million in August 2020 to 13.84 million in December 2020; the increases in cases varied across counties and states.^4^

However, the association of limited in-person attendance in football games with the spread of COVID-19 in the hosting counties is not well understood. Despite the recent availability of COVID-19 vaccines, strict restrictions on holding large sporting events may remain in place until a herd immunity is achieved. Hence, quantification of the association of in-person football games attendance with the spread of COVID-19 could inform appropriate management decisions in future.

The purpose of this study was to assess whether the NFL and NCAA football games with limited in-person attendance were association with substantially increased COVID-19 cases in the counties where they were held. We used a matching method and a difference-in-differences estimator suited for time-series, cross-sectional data to estimate the association of in-person attendance with the spread of new reported COVID-19 cases per 100 000 residents.

## Methods

This cross-sectional study used publicly available deidentified data and therefore did not require approval from an institutional review board or informed consent per 45 CFR 46.102. This study followed the Strengthening the Reporting of Observational Studies in Epidemiology (STROBE) reporting guideline.

### Data

We extracted NFL and NCAA game information, COVID-19 case counts, and COVID-19 policies and interventions from public sources. We gathered information on NFL and NCAA games played in the 2020 to 2021 regular season, spanning August 29, 2020, to December 28, 2020, from Pro Football Reference.^5^ Extracted data included game dates, in-person attendance (yes or no), and the stadiums where the games were held. We obtained stadiums’ longitude and latitude from Google Maps.^6^ Home stadiums’ longitude and latitude were mapped to the corresponding US county. We collected COVID-19 statewide nonpharmacological interventions and policies from the COVID-19 US State Policy Database with respect to physical distance closures (closing nonessential businesses and closing restaurants except take-out), stay-at-home orders (stay-at-home or shelter-in-place orders and ending or relaxing stay-at-home or shelter-in-place orders), and second closures and reopening (closing bars, reopening bars, reopening restaurants, and reopening nonessential businesses).^7^ We obtained county-level population data from the 2019 US Census Bureau Gazetteer Files.^8^ We computed county-level new cases of COVID-19 per 100 000 residents using data from the COVID-19 Data Repository by the Center for Systems Science and Engineering at Johns Hopkins University.^9^

### Statistical Analysis

We quantified the association of interest by comparing daily changes in COVID-19 cases per 100 000 residents in counties that have held NFL or NCAA games with limited in-person attendance with those that did not hold NFL or NCAA games or had no attendance. This time series cross-sectional study used matching and a difference-in-differences design to estimate the Average Treatment Effect on the Treated (ATT).^10^ The overall study design consisted of 3 major parts: constructing initial matched set for each treated county based on history of games, refinement of the matched sets based on additional control variables, and ATT estimation using a difference-in-differences estimator.

Matching methods applied to time-series cross-sectional data have been somewhat limited. A 2020 study by Imai et al^10^ noted that most researchers have used 2-way fixed effects regression for causal inference with time-series data. A notable exception is a 2009 study by Nielsen and Sheffield^11^ that used matching for time-series data, but the authors acknowledged that their algorithm was still in development. It is also significantly different than the adopted approach in this study, proposed by Imai et al,^10^ which builds on foundational methodological work by Abadie et al^12^ on matching and Robins et al^13^ on marginal structural models.

The binary treatment variable, *X_it_*, was defined as whether county *i* had any NFL or NCAA games with in-person attendance on date *t*. If there was a game with in-person attendance in county *i* at date *t*, *X_it_* was set to 1. If not, *X_it_* was set to 0. We defined posttreatment period *F* as the time period (days) after treatment, which we set to 14 days owing to the incubation period of coronaviruses.^14^ Similarly, we defined pretreatment period *L* to be 14 days. The outcome of interest was the change in new, reported daily COVID-19 cases per 100 000 residents from time *t* to time *t* + *F*, that is from day 0, *t_0_*, to day 14, *t_14_*.

### Constructing Initial Matched Set for Each Treated County

For each treated county *X_it_*, a set of matched counties was determined based on pretreatment and posttreatment game history, such that matched counties should have identical game history from time *t* – *L* to *t* *–* 1. Note that *t* is fixed for both treated and matching counties, so that with the same time trend, we can adjust for time-specific unobserved confounders later. Second, matched control units were excluded if they had a game with in-person attendance after time *t* but before the outcome is measured at time *t* + *F*. For each treated county *X_it_* with *X_it_* = 1 and *X_i,t_* – 1 = 0, the matched set of counties is defined as:


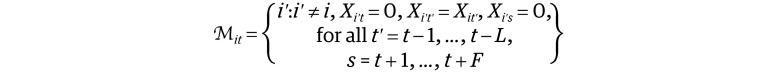


**Figure 1.**
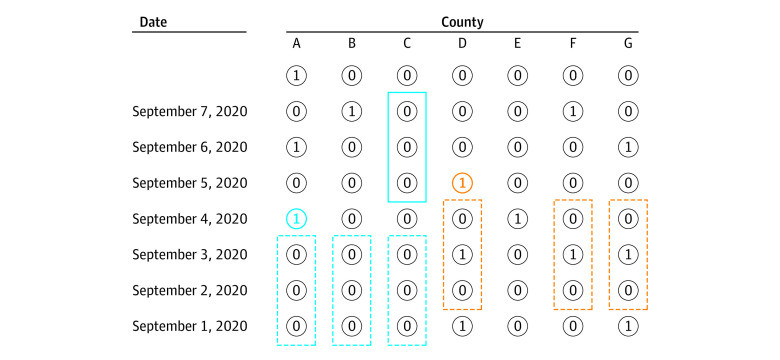
Illustration of Matched County Set With Pretreatment and Posttreatment Period Set at 3 Days Blue (county with match) and orange (county without match) circles represent treatment observations. For each treatment, counties with the exact same pretreatment history as the treated county are first selected (dashed rectangles). The selected counties get assigned to the matched set if their posttreatment periods have no games (solid rectangles). Circled zeroes indicate counties without games on that day, and circled 1s mean at least 1 game happened.

Figure 1 provides an illustration of the matching procedure. For illustration purposes, assume both pretreatment *L* and post-treatment *F* are equal to 3 (days). For the bolded blue circle, there is a treatment in county A on September 4, 2020. In the first matching step, both county B and C are selected because they have the same treatment history as county A (selected with dash-dotted rectangles). Though county E also has the same history, it is not selected, because it has treatment on September 4. In the next matching step, county B is filtered out because it has a treatment within 3 days after September 4. Hence, only county C is in the matched set of bolded blue treatment (with solid rectangles). In the next matching step, county B is filtered out because it has a treatment within 3 days after September 4. Hence, only county C is in the matched set of blue treatment (with solid rectangles). Similar matching procedures lead to no matched counties for bolded orange treatment. In brief, counties are matched counties only if they first have dashed rectangles around past history and then have solid rectangles around future events.

### Refinement of the Matched Sets Based on Additional Controls

In the initial matching step based on history of games played, other control variables that may play an important role in spread of the disease are ignored. However, in addition to the identical history of games, we also need to ensure that treatment and control groups are similar based on factors that may influence the disease epidemiological characteristics, such as population size and presence of nonpharmaceutical interventions, because otherwise, the required parallel trends assumption for difference-in-differences would not hold. In this step, we refine the matching algorithm to account for such control variables and ensure the validity of parallel trends assumption.

Every treated county *X_it_* has up to 10 control counties selected from the matched set *M_it_* with replacement. To this end, we use the mean Mahalanobis measure to calculate the distances between the treated county and each matched county over time, adjusting for covariates.^15,16^ As specified, covariates included COVID-19 statewide nonpharmacological interventions, policies, and county-level population. More specifically, nonpharmaceutical interventions and policies were extracted from the COVID-19 US State Policy database, which tracks when each state implemented and ended policies. Thus, it is a vector of features that captures intervention and policy changes on a daily basis. We added the following nonpharmaceutical interventions and policies to our time-series panel data after crosswalking them to their respective county: physical distance closures (closing nonessential businesses and closing restaurants except take-out), stay-at-home orders (stay-at-home or shelter-in-place and end or relax of stay-at-home or shelter-in-place), second closures and reopening (closing bars, reopening bars, reopening restaurants, and reopening nonessential businesses), as dates and binary (yes or no) variables. Additionally, we included county-level populations from the 2019 US Census Bureau Gazetteer Files and county-level new cases of COVID-19 per 100 000 residents using data from the Center for Systems Science and Engineering as numeric variables.

The mean Mahalanobis distance over pretreatment history in our study is defined as:
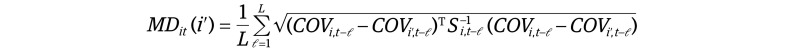
where *i’* ε M*_it_* is a matched county of *X_it_*, *COV_it_* is the time-varying covariates that we want to adjust for, and *S_i,t_* is the covariance matrix of *COV_it_*. In other words, given a control county in the matched set, we computed the standardized distance using the time-varying covariates, and calculate the mean over time. For each treated county, we chose control counties with the top 10 smallest Mahalanobis distance, if there are any, and assign equal weights to control counties in the refined matched set. Doing so implies that up to 10 most similar control counties under Mahalanobis distance are selected for the treated county:
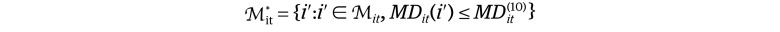
where
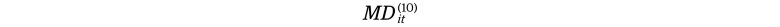
indicates the 10th smallest order statistic of *MD_it_ (i’)* among the original matched set *M_it_.*

### ATT Estimation

After the refined matched sets were obtained, we calculated the counterfactual outcome using the weighted average of control counties in M^*^_it_. For *K* treated counties and *T* observation days, the estimated ATT of the occurrence of a football game is defined as:
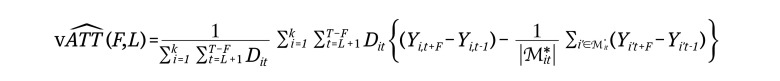
where outcome variable *Y_i,t_* is the new daily cases per 100 000 residents in county *i* on day *t*, and *D*_it_ = 1{*X_it_* = 1} × 1{*X_i,t_*_ – 1_ = 0} × 1{|M^*^_it_| > 0} is an indicator for a treatment *X_it_* with nonempty matching set.

Besides the time-fixed effect, this model also accounts for county-level fixed effects as they are eliminated by the difference between (*Y_i,t + F_* – *Y_i,t − 1_*) and (*Y_i’,t + F_* – *Y_i’,t − 1_*). To compute the SE of estimator
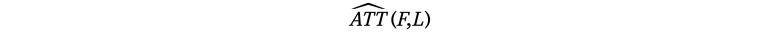
we used a block-bootstrap procedure designed for matching with time series cross-sectional data by conditioning on the weight. The number of bootstrap iterations was set to 1000.

We also performed sensitivity analyses by varying the pretreatment period and posttreatment period. All preprocessing of data and statistical analyses were performed using R statistical software version 4.0.3 (R Project for Statistical Computing). Data were analyzed from August 29 to December 28, 2020.

## Results

Out of 796 NFL and NCAA games (101 NFL games [19.1%]; 427 NCAA games [80.9%]) played from August 29, 2020, to December 28, 2020, 528 games (66.3%) had in-person attendance. The median (interquartile range [IQR]) attendance at NFL games during this period was 9949 (6000 to 13 797) people. NCAA game attendance numbers were not publicly available, and in person attendance was considered as a binary variable.

**Figure 2.**
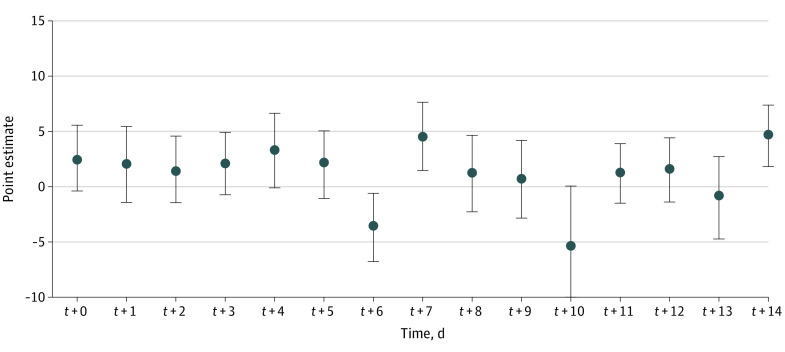
Estimated Average Treatment Effect Sizes Over Time Error bars indicate SEs.

The matching algorithm returned 361 matching sets. Covariate balance in the pretreatment time period between treated and matched control counties was assessed by computing the mean difference, measured as SD units, for each time-varying covariate in the pretreatment time period. The standardized mean difference for the outcome and the other time-varying covariates stayed relatively constant over the entire pretreatment period. Figure 2 shows the estimated ATT when posttreatment period *F* and pretreatment period *L* are set to 14 days, along with SE and 95% CI (eTable 1 in the Supplement). The median (IQR) daily new COVID-19 cases in treatment group counties hosting games was 26.14 (10.77-50.25) cases per 100 000 residents on game day. The median (IQR) daily new COVID-19 cases in control group counties where no games were played was 24.11 (9.64-48.55) cases per 100 000 residents on game day. These results suggest that the ATT ranged from −5.17 to 4.72, with a mean (SD) of 1.21 (2.67) cases per 100 000 residents within the 14-day period in all counties hosting the games, and the daily treatment effect trend remained relatively steady during this period.

**Figure 3.**
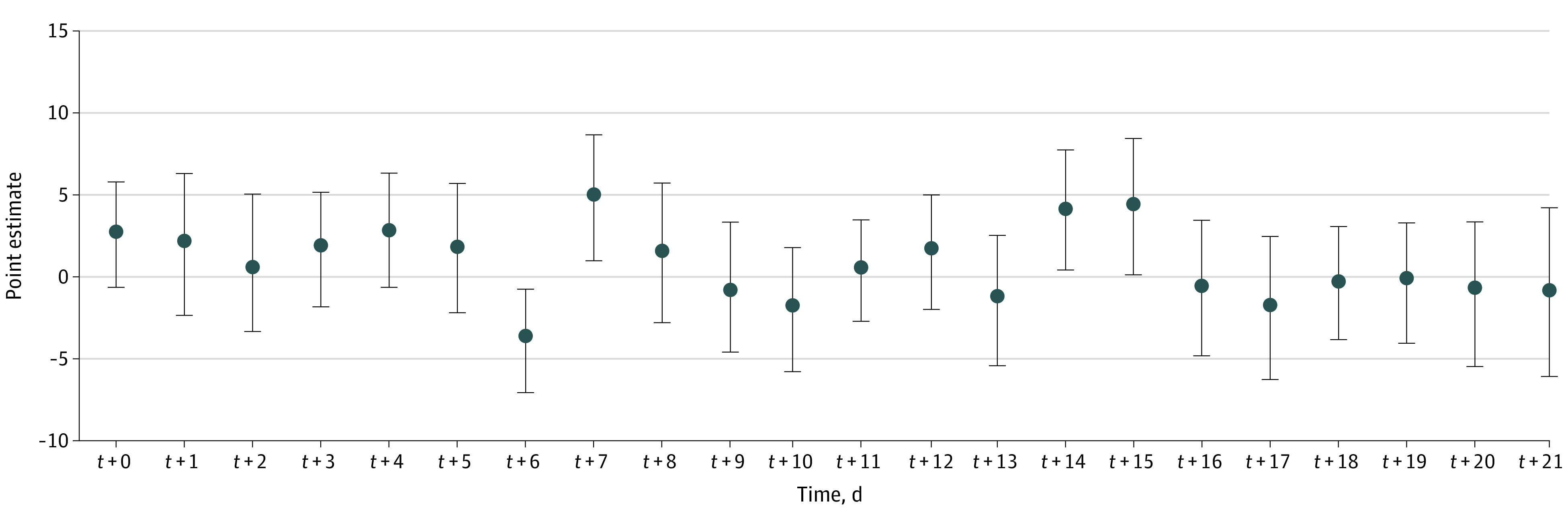
Robustness Check for the Estimated Average Treatment Effect Sizes Over Time Error bars indicate SEs.

To evaluate the robustness of our results, we performed sensitivity analyses by varying the pretreatment period and posttreatment period parameters *(L, F)* to (14, 7), (14, 14), (14, 7) and (21, 14) days. Across the different *(L, F)* parameter specifications, we found that the ATT remained similar to the baseline analysis (Figure 3; eTable 2 in the Supplement).

## Discussion

In this time-series, cross-sectional study of US counties with NFL and NCAA football games, we used matching and a difference-in-differences estimator to assess the association of games with in-person attendance with county-level COVID-19 spread compared with games held without any fans. The study considered the association of both NFL and NCAA games with higher COVID-19 cases, since many counties had both an NFL and NCAA game in the pretreatment or posttreatment periods, hence the association of NFL games alone or NCAA games alone with higher COVID-19 cases could not be studied separately. Games with in-person attendance were matched with a set of control counties that shared the same game history from time *t* –* *14 to *t* – 1 days. We selected 14 days based on the literature that suggests that the incubation period for COVID-19 is 2 to 14 days.^14^ To control for posttreatment bias, we apply the idea of marginal structural models by excluding control counties that had a game with in-person attendance after time *t* but before the 14-day posttreatment period is complete. We found that the ATT of in-person attendance at NFL and NCAA games on new, daily reported COVID-19 cases per 100 000 residents was not substantial over a 14-day period. We surmise that the NFL and NCAA policies regarding limited in-person attendance, mask use, and social and physical distancing measures in stadiums was not associated with substantially higher community spread of COVID-19.^17,18^ Additionally, an important number of NFL and NCAA football stadiums are outdoors or have a retractable roof, which could have had an impact on mitigating spread.^19^

### Limitations

There are limitations to this study. First, owing to data limitations, we considered in-person attendance as a 0 or 1 binary variable. Specifically, while in-person attendance numbers were available for NFL games, they were not available for NCAA games. Explicit consideration of attendance numbers may change the estimation. Second, the Stable Unit Treatment Value Assignment assumption imposed that each unit receives the same form or version of the treatment.^20^ Third, we did not control for other large gathering events, such as political rallies, although some of these types of events have been found to be associated with a local increase in COVID-19 cases.^21^ Third, we also did not account for the spillover effects to the counties adjacent to the ones hosting NFL or NCAA games. Fourth, to account for heterogeneity in county characteristics, we controlled for physical distance closures (closing nonessential businesses and closing restaurants except take-out), stay-at-home orders (stay-at-home or shelter-in-place and end or relax of stay-at-home or shelter-in-place), second closures and reopening (closing bars, reopening bars, reopening restaurants, and reopening nonessential businesses), and county-level populations. However, despite controlling for these factors, potential inherent heterogeneity across counties remains as a limitation.

## Conclusions

This cross-sectional study provides new information on the association of football games with in-person attendance with COVID-19 spread. The findings of this time-series, cross-sectional study with difference-in-differences design suggest that NFL and NCAA games held with limited in-person attendance were not associated with increased COVID-19 cases in the counties where they were held. Further research is needed to account for potential spillover to counties adjacent to the those hosting games. While COVID-19 vaccination has started, restrictions will likely stay in place for several months and possibly until the start of the 2021 to 2022 NFL and NCAA seasons. Our study provides evidence suggesting that in-person attendance of football games with social distancing and mask use could be resumed in the 2021 to 2022 season. However, it is worth noting that newly emerging variants of SARS-CoV-2 have less predictable implications at this point and might lead to more disruptive interruptions in the future.

## Notes

Supplement. **eTable 1.** Estimated Average Treatment Effects of NFL and NCAA Football Games With In-Person Attendance on the Spread of COVID-19 Over Time With Pretreatment and Posttreatment Periods set at 14 Days **eTable 2.** Estimated Average Treatment Effects of NFL and NCAA Football Games With In-Person Attendance on the Spread of COVID-19 Over Time With Pretreatment and Posttreatment Periods set at 21 Days

## References

[1] Centers for Disease Control and Prevention. Guidance for organizing large events and gatherings. 2020. Accessed July 2, 2021. https://www.cdc.gov/coronavirus/2019-ncov/community/large-events/considerations-for-events-gatherings.html

[2] The New York Times. See reopening plans and mask mandates for all 50 states. *The New York Times*. Updated July 1, 2021. Accessed July 2, 2021. https://www.nytimes.com/interactive/2020/us/states-reopen-map-coronavirus.html

[3] McCloskey B, Zumla A, Ippolito G, ; WHO Novel Coronavirus-19 Mass Gatherings Expert Group. Mass gathering events and reducing further global spread of COVID-19: a political and public health dilemma. Lancet. 2020;395(10230):1096-1099. doi:10.1016/S0140-6736(20)30681-4 32203693 PMC7138150

[4] Ritchie H, Ortiz-Ospina E, Beltekian D, ; Our World in Data. Coronavirus pandemic (COVID-19). Accessed July 2, 2021. https://ourworldindata.org/coronavirus

[5] Pro Football Reference. 2020 NFL attendance data. Accessed July 2 2021. https://www.pro-football-reference.com/years/2020/attendance.htm

[6] Google Maps. Accessed July 2, 2021. https://www.google.com/maps

[7] Raifman J, Nocka K, Jones D, , COVID-19 US state policy database. Accessed July 2, 2021. https://www.openicpsr.org/openicpsr/project/119446/version/V109/view

[8] US Census Bureau. Gazetteer Files. 2020; Accessed July 2, 2021. https://www.census.gov/geographies/reference-files/time-series/geo/gazetteer-files.html

[9] Dong E, Du H, Gardner L. An interactive web-based dashboard to track COVID-19 in real time. Lancet Infect Dis. 2020;20(5):533-534. doi:10.1016/S1473-3099(20)30120-1 32087114 PMC7159018

[10] Imai K, Kim IS, Wang E. Matching methods for causal inference with time-series cross-sectional data. Accessed July 2, 2021. https://imai.fas.harvard.edu/research/tscs.html

[11] Nielsen R, Sheffield J. Matching with time-series cross-sectional data. Accessed July 2, 2021. http://citeseerx.ist.psu.edu/viewdoc/summary?doi=10.1.1.510.7097

[12] Abadie A, Diamond A, Hainmueller J. Synthetic control methods for comparative case studies: estimating the effect of California’s tobacco control program. J Am Stat Assoc. 2010;105(490):493-505. doi:10.1198/jasa.2009.ap08746

[13] Robins JM, Hernán MA, Brumback B. Marginal structural models and causal inference in epidemiology. Epidemiology. 2000;11(5):550-560. doi:10.1097/00001648-200009000-00011 10955408

[14] Centers for Disease Control. Clinical questions about COVID-19: questions and answers. Updated March 4, 2021. Accessed July 2, 2021. https://www.cdc.gov/coronavirus/2019-ncov/hcp/faq.html#:~:text=Based%20on%20existing%20literature%2C,2–14%20days

[15] Stuart EA. Matching methods for causal inference: a review and a look forward. Stat Sci. 2010;25(1):1-21. doi:10.1214/09-STS313 20871802 PMC2943670

[16] Fredriksson A, de Oliveira GM. Impact evaluation using difference-in-differences. RAUSP Manage J. 2019;54(4):519-532. doi:10.1108/RAUSP-05-2019-0112

[17] Traub M. How many fans will be at college football games this year? *SportsTravel*. December 14, 2020; Accessed July 2, 2021. https://www.sportstravelmagazine.com/college-football-2020-schedule-fans-attend-status-sec-acc-big-12-covid-19-pandemic/

[18] ESPN NFL Nation. Where each of the 32 NFL teams stands on allowing fans into stadiums. *ESPN*. December 14, 2020. Accessed July 2, 2021. https://www.espn.com/nfl/story/_/id/29910246/where-32-nfl-teams-stands-allowing-fans-stadiums

[19] Centers for Disease Control and Prevention. Guidance for unvaccinated people: deciding to go out. Updated October 2020. Accessed July 2, 2021. https://www.cdc.gov/coronavirus/2019-ncov/daily-life-coping/deciding-to-go-out.html

[20] Imbens GW Rubin DB. Causal Inference for Statistics, Social, and Biomedical Sciences: An Introduction. Cambridge University Press; 2015. doi:10.1017/CBO9781139025751

[21] Bernheim DB, Buchmann N, Freitas-Groff Z, Otero S. The effects of large group meetings on the spread of COVID-19: the case of Trump rallies. *Stanford Institute for Economic Policy Research*. October 2020. Accessed July 2, 2021. https://siepr.stanford.edu/research/publications/effects-large-group-meetings-spread-covid-19-case-trump-rallies

