# Supplementary material for: Association of Limited In-Person Attendance in US National Football League and National Collegiate Athletic Association Games With County-Level COVID-19 Cases
Source: JAMA Netw Open. 2021 Aug 17;4(8):e2119621. doi: 10.1001/jamanetworkopen.2021.19621 (PMC8371570; doi:10.1001/jamanetworkopen.2021.19621)
Supplement: Supplement. — eTable 1. Estimated Average Treatment Effects of NFL and NCAA Football Games With In-Person Attendance on the Spread of COVID-19 Over Time With Pretreatment and Posttreatment Periods set at 14 Days eTable 2. Estimated Average Treatment Effects of NFL and NCAA Football Games With In-Person Attendance on the Spread of COVID-19 Over Time With Pretreatment and Posttreatment Periods set at 21 Days [file jamanetwopen-e2119621-s001.pdf]

# Supplemental Online Content

Toumi A, Zhao H, Chhatwal J, Linas BP, Ayer T. Association of limited in-person attendance in US National Football League and National Collegiate Athletic Association games with county-level COVID-19 cases. *JAMA Netw Open*. 2021;4(8):e2119621. doi:10.1001/jamanetworkopen.2021.19621

**eTable 1.** Estimated Average Treatment Effects of NFL and NCAA Football Games With In-Person Attendance on the Spread of COVID-19 Over Time With Pretreatment and Posttreatment Periods set at 14 Days
**eTable 2.** Estimated Average Treatment Effects of NFL and NCAA Football Games With In-Person Attendance on the Spread of COVID-19 Over Time With Pretreatment and Posttreatment Periods set at 21 Days

This supplemental material has been provided by the authors to give readers additional information about their work.

© 2021 Toumi A et al. *JAMA Network Open.*

**eTable 1.** Estimated Average Treatment Effects of NFL and NCAA Football Games With In-Person Attendance on the Spread of COVID-19 Over Time With Pretreatment and Posttreatment Periods set at 14 Days

| **Time (days)** | **ATT** | **Standard error** | **2.5% percentile** | **97.5% percentile** |
|---|---|---|---|---|
| t+0 | 2.430 | 1.52 | -0.389 | 5.57 |
| t+1 | 2.055 | 1.69 | -1.46 | 5.45 |
| t+2 | 1.396 | 1.56 | -1.43 | 4.57 |
| t+3 | 2.088 | 1.46 | -0.727 | 4.91 |
| t+4 | 3.329 | 1.67 | -0.105 | 6.62 |
| t+5 | 2.179 | 1.58 | -1.06 | 5.04 |
| t+6 | -3.535 | 1.54 | -6.76 | -0.588 |
| t+7 | 4.522 | 1.56 | 1.45 | 7.64 |
| t+8 | 1.258 | 1.77 | -2.28 | 4.62 |
| t+9 | 0.706 | 1.79 | -2.83 | 4.18 |
| t+10 | -5.176 | 2.89 | -10.1 | 0.0656 |
| t+11 | 1.294 | 1.40 | -1.5 | 3.9 |
| t+12 | 1.623 | 1.49 | -1.36 | 4.41 |
| t+13 | -0.789 | 1.85 | -4.73 | 2.72 |
| t+14 | 4.722 | 1.43 | 1.83 | 7.39 |

*The average treatment effects on the treated (ATT), standard errors (SE) and 95% confidence intervals are shown over the post-treatment period from $t_0$ to t+14 days.*

© 2021 Toumi A et al. *JAMA Network Open.*

**eTable 2.** Estimated Average Treatment Effects of NFL and NCAA Football Games With In-Person Attendance on the Spread of COVID-19 Over Time With Pretreatment and Posttreatment Periods set at 21 Days

| **Time (days)** | **ATT** | **Standard error** | **2.5% percentile** | **97.5% percentile** |
|---|---|---|---|---|
| t+0 | 2.777 | 1.72 | -0.626 | 5.83 |
| t+1 | 2.196 | 2.12 | -2.35 | 6.32 |
| t+2 | 0.550 | 2.18 | -3.36 | 5.07 |
| t+3 | 1.936 | 1.83 | -1.86 | 5.14 |
| t+4 | 2.861 | 1.75 | -0.664 | 6.34 |
| t+5 | 1.848 | 2.03 | -2.23 | 5.71 |
| t+6 | -3.610 | 1.62 | -7.1 | -0.732 |
| t+7 | 5.064 | 1.93 | 0.964 | 8.69 |
| t+8 | 1.583 | 2.20 | -2.84 | 5.71 |
| t+9 | -0.778 | 2.00 | -4.64 | 3.36 |
| t+10 | -1.737 | 1.97 | -5.81 | 1.8 |
| t+11 | 0.554 | 1.57 | -2.71 | 3.49 |
| t+12 | 1.731 | 1.76 | -1.99 | 5.01 |
| t+13 | -1.176 | 2.04 | -5.44 | 2.55 |
| t+14 | 4.181 | 1.85 | 0.426 | 7.75 |
| t+15 | 4.448 | 2.13 | 0.127 | 8.45 |
| t+16 | -0.553 | 2.19 | -4.87 | 3.49 |
| t+17 | -1.746 | 2.25 | -6.31 | 2.45 |
| t+18 | -0.275 | 1.76 | -3.83 | 3.05 |
| t+19 | -0.066 | 1.92 | -4.1 | 3.25 |
| t+20 | -0.675 | 2.20 | -5.5 | 3.4 |
| t+21 | -0.814 | 2.56 | -6.05 | 4.2 |

*The average treatment effects on the treated (ATT), standard errors (SE) and 95% confidence intervals are shown over the post-treatment period from $t_0$ to t+21 days.*

© 2021 Toumi A et al. *JAMA Network Open.*
